# Toxicity Analysis of Hybrid Nanodiamond/Fe_3_O_4_ Nanoparticles on *Allium cepa* L

**DOI:** 10.1155/2022/5903409

**Published:** 2022-09-19

**Authors:** Bhaludra Chandra Sekhar Singh

**Affiliations:** Department of Plant Science, College of Agriculture and Veterinary Sciences, Guder Mamo Mezemir Campus, Ambo University, Ambo, P.O. 19, Ethiopia

## Abstract

**Materials and Methods:**

The chemicals of hydrochloric acid, nitric acid, FeCl_3_.6H_2_O, FeCl_2_.4H_2_O, NaCl, and NaOH (Sigma-Aldrich chemicals, USA) were utilized in this study. A statistical analysis was performed on the results with a prevalence of *p* < 0.05.

**Results:**

A novel ND/Fe_3_O_4_ nanocomposite material was successfully synthesized by the *in-situ* method and characterized by various characterization techniques. The analysis of X-ray diffraction indicated the formation of an ND/Fe_3_O_4_ nanocomposite with both participating phases. The saturation magnification of the ND/Fe_3_O_4_ nanocomposite is 13.2 emu/g, whereas for a pure Fe_3_O_4_ nanomaterial, it is 47 emu/g. The weight rates of ND and Fe_3_O_4_ existent in the nanocomposite are 28% and 72%, respectively. From the electrical conductivity analysis, ND/Fe_3_O_4_ exhibits conductivity in the order of 27 times more compared to ND.

**Conclusion:**

The result implies that the product ND/Fe_3_O_4_ has both magnetic and electrical properties. The biocompatibility of the synthesized ND/Fe_3_O_4_ material was studied based on the *in-vitro* method.

## 1. Introduction

Nanocomposites containing two phases of materials in the nanometer range of(<100 nm) are a critical part of nanotechnology and the active growing areas in materials science and engineering. Solid coupling between various combining parts results in novel physical wonders and improved prospects, which makes these frameworks better than their single-segment partners for application in biomedicine, nanoelectronics, optoelectronics, and spintronics applications [1]. Nanodiamond particles (ND) with a size of 4–5 nm demonstrate an extraordinary powerful application in fluorescence markers, restorative items, polymers, biosensors, and medication transmission [2–4]. The ND particles are normally combined with carbon-polluting influences (size of 100–200 nm), which is not possible in particular applications. Deaggregation of ND nanoparticles from micron-level to nano-level is essential. A nanostructured ND composite with various materials was synthesized by different techniques. Shi et al. [5] combined ND/Cu nanocomposites for synergist applications. Nunes et al. [6] investigated Ni/ND and Ni/graphite composites delivered by mechanical combination and warmth treatment strategy. Bahadar et al. [7] studied cell toxicity, genotoxicity, and immunotoxicity. Sundar et al. [8] orchestrated ND-Ni nanocomposites by using the in-situ strategy and arranged nanocomposite nanofluids for warm applications. Kumar et al. [9] analyzed the drilling execution of nanodiamond bonded SiC composites for CFRRP laminates by a dual-axis grinding wheel system. Abakumov et al. [10] performed high biocompatibility of obtained nanoparticles and the number of in-vitro toxicological tests on human fibroblasts and U251 glioblastoma cells. It was shown that albumin nanoparticles' coating provides a stable and biocompatible shell and prevents cytotoxicity of the magnetite core. On long exposure times (48 hours), cytotoxicity of iron oxide nanoparticles takes place due to free radical production, but this toxic effect may be neutralized by using polyethylene glycol modification. Bao et al. [11] used CuO nanoparticles to study the toxicity using various *Saccharomyces cerevisiae* (*S*. *cerevisiae*) strains, and wild type, single-gene-deleted mutants, and multiple-gene-deleted mutants, were determined and compared. Assadian et al. stated that [12] CuO-NPs have been employed in the pharmaceutical industry, especially in the production of antimicrobial fabric treatments or the prevention of infections caused by *Escherichia coli* and methicillin-resistant *Staphylococcus aureus*. Two key potential routes of exposure to CuO-NPs exist through inhalation and skin exposure. Ozgur et al. [13] studied the toxicity analysis of Fe_3_O_4_ nanoparticles on the superoxide dismutase (SOD) and catalase (CAT) activities which showed a significant (*p* < 0.05) decrease after 100 mg/L after exposure to Fe_3_O_4_ NPs for 24 h. As the doses of Fe_3_O_4_ NPs increased, the levels of malondialdehyde (MDA) and total glutathione (tGSH) significantly (*p* < 0.05) increased at doses of 400 and 800 mg/L, respectively.

The above-mentioned works are related to ND nanocomposites with magnetic and nonmagnetic materials. There are several applications with magnetic materials such as Fe_2_O_3_, Fe_3_O_4,_ and Ni especially in drug delivery, MRI contrast agent, and magnetic hyperthermia. [14]. Under atmospheric conditions, the magnetization of Fe_3_O_4_ is high compared to the magnetization of Fe_2_O_3_ [15, 16]. Huang et al. [17] explained the emphasis of nanoparticles that influence cytotoxicity. Identification of those properties may lead to the design of more efficient and safer nanosized products for various industrial purposes and guide the assessment of human and environmental health risks. Rotini et al. [18] studied the toxicity of CuO nanoparticles and observed the physical interactions between *Vibrio* and CuO nanoparticles. Cando et al. [19] studied the neurotoxicity, and they observed mitochondrial activity starting at 10 *μ*g·mL^−1^ with a decrease in cellular vitality of 35% and a maximum decrease of 45% at the highest dose (100 *μ*g·mL^−1^).

However, to the best of my knowledge, nanocomposites made of ND with magnetic Fe_3_O_4_ have not been intensively investigated so far. This work aims to prepare a high purity ND/Fe_3_O_4_ nanocomposite and estimate its magnetic and biocompatibility properties.

## 2. Materials and Methods

### 2.1. Study Area

The study was executed at the Department of Genetics & Biotechnology, Osmania University, from January 2020 to March 2021. The plant material and nanoparticles were obtained from the same institution.

### 2.2. Chemicals

The following chemicals such as hydrochloric acid, nitric acid, FeCl_3_.6H_2_O, FeCl_2_.4H_2_O, NaCl, NaOH (Sigma-Aldrich chemicals, USA), and distilled water (Milli-Q) were used. The exploded ND ash was acquired from ITC with virtue (>98%; cubic stage; 4–5 nm molecule estimates).

### 2.3. Deaggregation of ND Particles

Commercially available ND soot aggregates with amorphous carbon impurities of microbe size. It is important to deaggregate and purify these ND particles into almost single ND crystals. For decollection, ND ash was broken up in a fluid NaCl arrangement and pursued by tip sonication for up to 5 hrs, separated and washed a few times with refined water, and after that solidified and dried. To functionalize and remove nondiamond carbon impurities, the deaggregated ND soot was treated with a strongly acidic medium containing 1 : 3 molar ratios of hydrochloric corrosive and nitric corrosive [20] for up to 72 hours under attractive mixing at 60°C. Then, the particles were washed a few times with refined water and dried in the stove at 80°C for up to 24 hrs. The benefit of this method is the removal of nondiamond carbon impurities and the formation of carboxyl bunches on the surface of the ND molecule.

### 2.4. Preparation of the ND/Fe_3_O_4_ Magnetic Composite

The ND/Fe_3_O_4_ composite was prepared by the *in-situ* method. This method includes a dispersion of 0.3 g of treated ND in 30 m of refined water under attractive mixing for 60 minutes. After that, FeCl_3_.6H_2_O (0.33 g) and FeCl_2_. 4H_2_O (0.165 g) were added in the molar ratio of 2 : 1. After full dispersion of iron salts, an aqueous NaOH solution was added drop by drop, and the arrangement pH was kept at 12. After vigorous stirring, the formation of a black coloured solution was observed. It indicates the reaction was completed, and the resulting black precipitate was washed a few times with refined water and dried in a broiler at 80°C for 24 h. The same procedure was used to synthesize the Fe_3_O_4_ nanoparticles without treating ND in distilled water for comparison purposes.

### 2.5. Characterization Techniques

The ND/Fe_3_O_4_ composite was portrayed using an X-beam diffractometer (Siemens D-500, 45 kV, and 40 mA), SEM (Hitachi; SU-70), and micro-Raman (Jobin-Yvon LabRam; 514 nm argon-ion laser). FTIR spectra were recorded using a Bruker Equinox V70 FTIR spectrometer in dry KBr pullet in the scope of 400–4000 cm^−1^ and atomic power microscopy (AFM, NT-MDT, NTEGRA Aura, and Nanotec's AFM with Dulcinea Electronic). The immersion polarization of the composite was investigated using a vibrating test magnetometer (VSM), Cryogenic, UK.

### 2.6. Bio Toxicity Study

#### 2.6.1. *Allium cepa* L. Culture, Root Tip Collection, and Exposure Conditions

The round fully grown onions were used for the analysis, and they were washed with water before they were used in the equipment. The temperature of the onions were s maintained at 28 ± 0.5°C. Meristematic root tips of 2-3 cm were obtained with a sharp cutting-edge knife. The square root tips are turned to different sides, those are placed in round-mouthed measuring glasses by using 5 *μ*g·mL^−1^ of Co_3_O_4_, 10 *μ*g·mL^−1^ of cND, and 20 *μ*g·mL^−1^ of cND-Co_3_O_4_, and those are treated with distilled water for 15 min. At around 4 h of time, the root tips were rinsed 3 times with water. The root tips were placed in a 5 m tube along with Cornoy's liquid (acidic corrosive: ethanol in a 1 : 3 ratio) to capture the dynamic mitosis. Root tips set in cylinders with Milli-Q water were treated as the control group, and three duplicates had been made for every target.

### 2.7. Root Tip Squash Preparation and Light Microscopy

The structure was adopted for the root tip squash planning and light microscopy, as shown by Kumari et al. in their illustration from [21]. In a nutshell, settled root tips were individually corrosive hydrolyzed in 0.1 N hydrochloric corrosive for 2 min at 60°C to degenerate solidifying material between the cells. After recoloring corrosive hydrolyzed pull tips with acetocarmine for 4–6 minutes, root tip squash was made by applying pressure to separate slides with coverslips. Using a light microscope, arranged slides that had accidentally been treated with nail polish were examined for the distinctive cyto-hereditary traits described in the current study. The number of separating cells per 1,000 observed cells was used to calculate the mitotic cells list, while alternative cells were also examined based on how many cells were uploaded and how many were scored in each combination.

### 2.8. Data Analysis

The mean and standard error (SE) of statistical analyses were verified, and Student's *t*-test was used to test for drift. At p 0.05, the degree of significance for each result was confirmed.

## 3. Results and Discussion

### 3.1. Characterization

**Figure 1:**
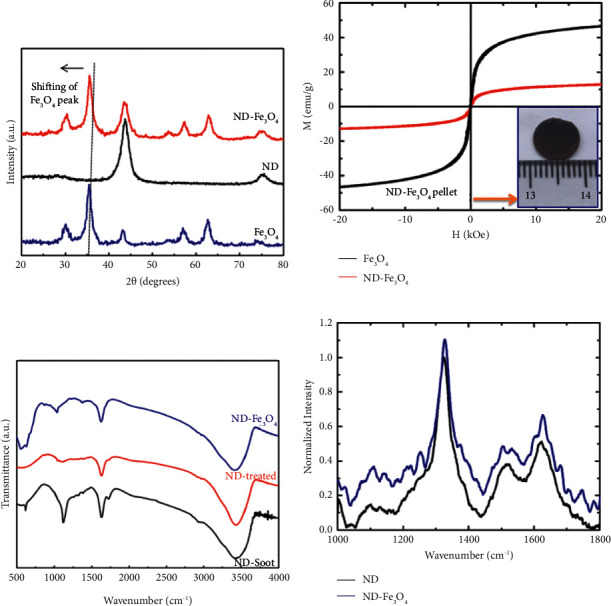
Characterization techniques. Footnote: (a) X-ray diffraction pattern. (b) M-H curves. (c) FTIR spectroscopy. (d) Raman spectroscopy.

The produced ND, Fe_3_O_4,_ and ND-Fe_3_O_4_ composites were examined by XRD analysis, and the patterns were presented in Figure 1(a). For ND, the 2*θ* values for (111) and (220) planes were 43.69 and 75.36  which match well with the JCPDS card (JCPDS NO: 00-006-0675), and no carbon-based impurity peaks were detected within the accuracy of XRD. For Fe_3_O_4,_ the 2*θ* values for (220), (311), (400), (511), (440), and (533) planes are 30.09 , 35.49 , 43.28 , 53.93 , 56.99 , 62.62 , and 74.02 , respectively; no other iron peaks were observed. The hkl values of all the peaks are provided. The (311) peak position of pure Fe_3_O_4_ is 35.5, which is shifted to 34.3 for the ND-Fe3O4 sample because of the presence of ND.

Table 1 demonstrates the X-ray diffraction positions (2*θ*) and the interplanar spacing values (d hkl) of the Fe_3_O_4_ sample (blue color line), Table 2 furnishes the X-ray diffraction positions (2*θ*) and the interplanar spacing values (d hkl) of the ND sample (black color line), and Table 3 shows X-ray diffraction positions (2*θ*) and the interplanar spacing values (d hkl) of the ND-Fe_3_O_4_ sample (red color line). The crystallite size of Fe_3_O_4_, ND, and ND-Fe_3_O_4_ was calculated from the Scherrer equation.

(1) $$\begin{array}{c} D=\frac{0.9\lambda }{\beta \text{cos}\theta }. \end{array}$$

*D* is crystalline size (nm), *λ* is wavelength (nm), *β* is full width at half maximum intensity peak in radians, and *θ* is the angle of diversion reciprocal to peak. The crystallite size of Fe_3_O_4_ was calculated from the high intensity peak, and it is noticed at 45.2 nm. The crystallite size of ND was calculated at a high-intensity peak, and it was observed as 6.96 nm. The final ND-Fe_3_O_4_ nanoparticles' crystalline size was calculated at a high-intensity peak, and it is found as 53.6 nm. The lattice constant of the Fe_3_O_4_ sample is 8.36 Å, whereas the lattice parameter of cubic nanodiamond is 3.55 Å. Similarly, the lattice parameter of the ND-Fe_3_O_4_ sample is 8.81 Å.

The magnetic hysteresis loops of Fe_3_O_4_ and ND/Fe_3_O_4_ composites at atmospheric temperature were shown in Figure 1(b). Mitra et al. [22] prepared amine-coated very small (6–14 nm) octahedral magnetite (Fe_3_O_4_) nanoparticles. Rathod et al. [23] prepared Fe_3_O_4_ and tested saturation magnetization and found it was 30 emu/g. For the Fe_3_O_4_ sample, the saturation magnetization is 47 emu/g, which corresponds to a particle size of 20 nm. For the ND/Fe_3_O_4_ composite sample, the saturation magnetization is 13.2 emu/g. This decline in mass polarization is expected for nonmagnetic ND particles present in the ND/Fe_3_O_4_ composite. Within the sight of the attractive field, the nonattractive particles go about as voids and break the attractive circuits, bringing about the decline of aggregate charge. The total magnetization for Fe_3_O_4_ is 47 emu/g and for ND-Fe_3_O_4_, it is 13.2 emu/g, which corresponds to 28 wt. (%) of Fe_3_O_4_ and 72 wt. (%) of ND. Comparative outcomes were accounted for in writing for the congregations of magnetic and nonmagnetic particles. Ghosh et al. [24] used the chemical coprecipitation method for the preparation of 12, 6, and 8 nm sides of Fe_3_O_4_, Fe_3_O_4_ closed with either polyethylene glycol (Fe_3_O_4_-PEG-MN) or oleic acid (Fe_3_O_4_-OA-MN).

**Figure 2:**
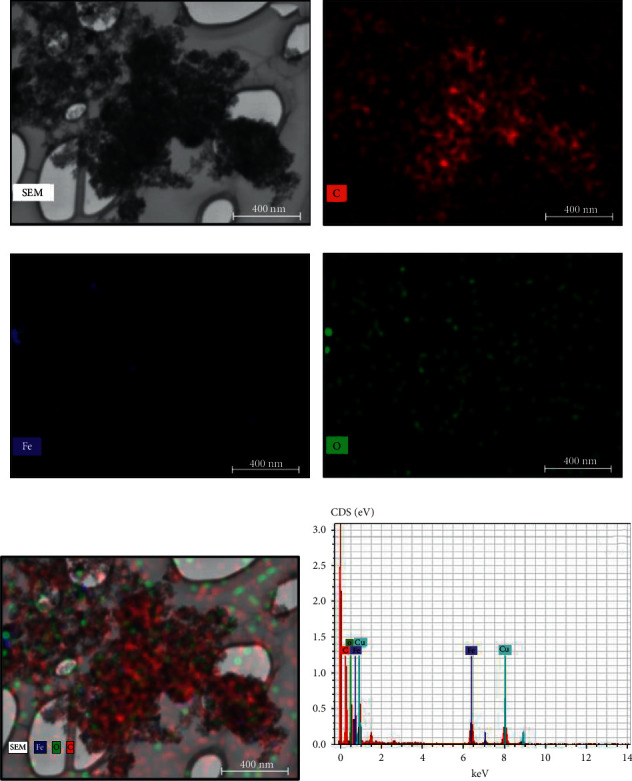
surface morphology and elemental mapping of the ND/Fe_3_O_4_ nanocomposite. Footnote: (a) SEM image of the ND/Fe_3_O_4_ composite on a copper grid and corresponding elemental mapping ((b)–(f)) energy-dispersive X-ray spectroscopy quantitative analysis.

**Figure 3:**
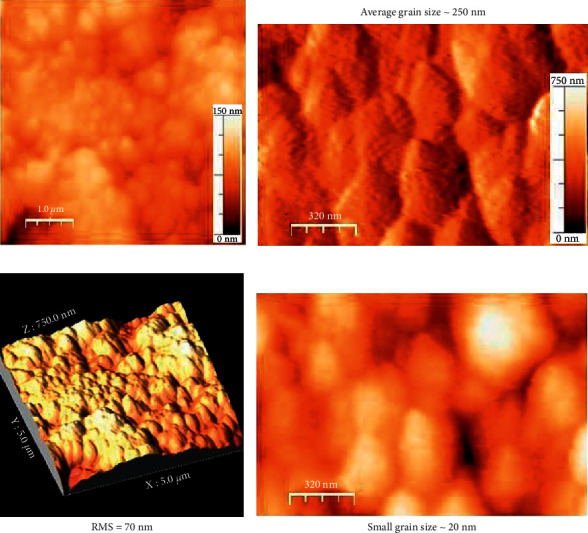
The surface topology of the ND/Fe3O4 composite from the atomic force microscopy: (a) general scan, (b) large grain size is 250 nm, (c) RMS of the hybrid nanoparticle is 70 nm, and (d) small grain size is 20 nm.

The FTIR spectra of ND soot and considered ND particles reveals that the peak at ∼1735 cm^−1^is ascribed to the C = O stretch of carboxylic (COOH) (Figure 1(c)), which is verification of a covenant connection of carboxylic gathering on the surface of the ND molecule. Notwithstanding, this pinnacle is not seen in the ND/Fe_3_O_4_ composite amid the in-situ development, which implies that the carboxylic gatherings get lessened amid the arrangement of Fe_3_O_4_ nanoparticles at first glance of ND.  Figure 1(d) shows the Raman spectra of treated ND and ND/Fe_3_O_4_ composites. A diamond peak in ND/Fe_3_O_4_ is observed at 1328 cm^−1^with a shoulder at 1130 cm^−1^ that starts from littler ND particles or littler rational dissipating spaces isolated by imperfections in bigger ND particles [8]. Another unmistakable component in the Raman spectra of the ND/Fe_3_O_4_ composite is a wide crest somewhere in the range of 1500–1700 cm^−1^, which is a superposition of the graphitic carbon band and the O-H band. Surface morphology, elemental mapping, and EDS analysis of the ND/Fe_3_O_4_ composite are shown in Figures 2(a)–2(f). The sample was arranged on a copper grid. From energy-dispersive X-ray spectroscopy, the ND/Fe_3_O_4_ composite contains 77.61 wt. (%) of carbon, 13.90 wt. (%) of oxygen, 0.19 wt. (%) of iron, and 8.30 wt. (%) of copper. Atomic force microscopy results of the ND/Fe_3_O_4_ composite were performed on 1 cm pellets.  Figure 3(a) indicates the general scan, Figure 3(b) shows the large grain size that is 250 nm, Figure 3(c) indicates the RMS of the hybrid nanoparticle that is 70 nm, and Figure 3(d) displays the small grain size which is 20 nm.

Each sample contains an amount of 80 mg powder and is made into a circular (diameter 1 cm; inset in Figure 1(a)) pellet for electrical measurements by using the four-point probe technique [25] which is typically used for highly conductive materials, where the contact resistance between sample and connection wires could play an important role, leading to a wrong estimate of the real material resistance [26]. To perform I–V measurements, a setup already tested was chosen and used for the estimation of ND soot, ND-treated, ND/Fe_3_O_4,_ and Fe_3_O_4_ samples at room temperature. To make the associations specifically onto our examples surface in a simple, quick, and reproducible way, the silver conductive glue was picked [27], which does not require specific gear, for example, metals sputtering or warm evaporator gadgets.

### 3.2. Biocompatibility

Once in ecological chambers, either emphatically or unintentionally, the destiny and effect of nanoparticles on real biota must be lit up, as this information can be an unequivocal factor in the success of nanotechnology. As already mentioned, the chromosomes of plants and living things have comparable morphology and comparative reactions to mutagens [28]. In this study, *Allium cepa* L. was used as a test model to clarify the potential cyto-genotoxicity of single nanoparticles of Fe_3_O_4_, cND, and the nanocomposite of cND- Fe_3_O_4_.

**Figure 4:**
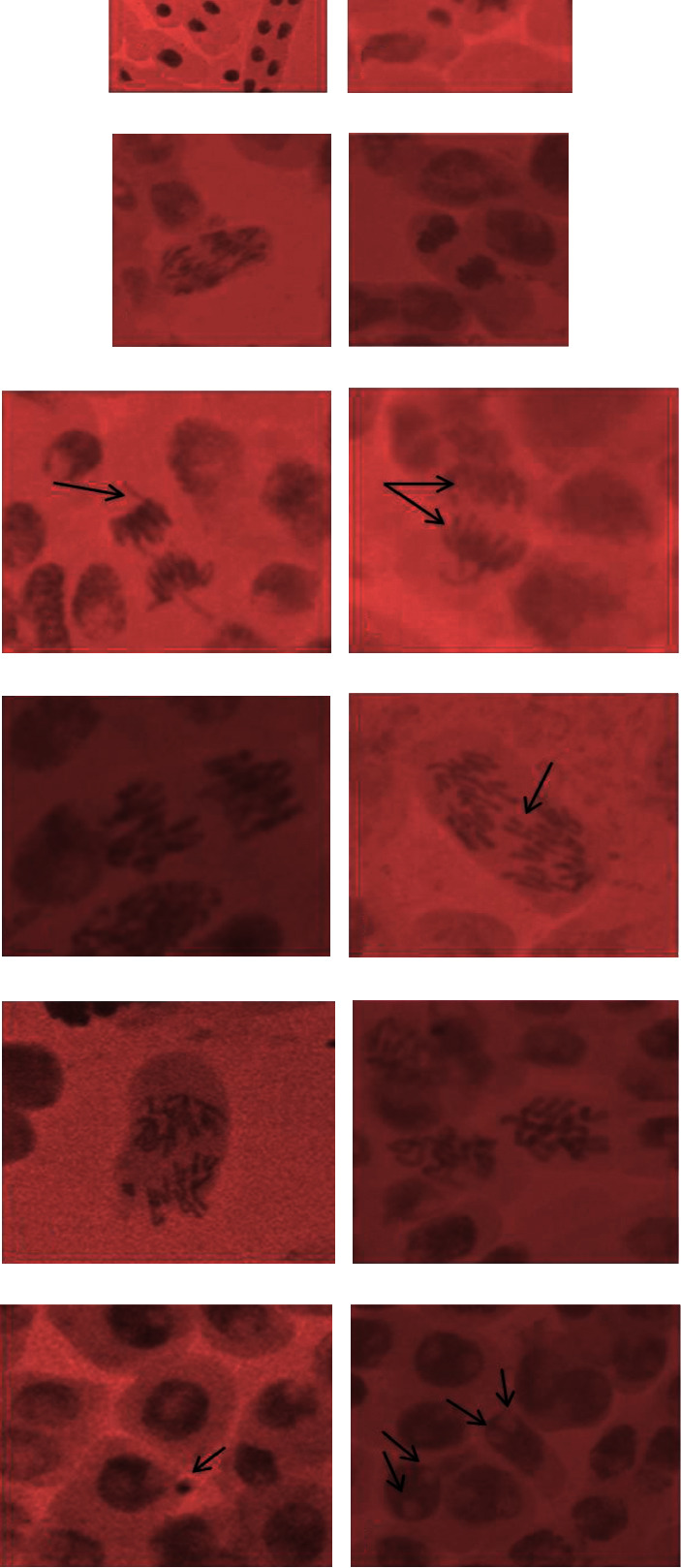
Representative images showing different chromosomal aberrations observed in *Allium cepa*. Footnote: *Allium cepa* root meristematic cells under control ((a)–(d)) and exposed to various concentrations of cobalt oxide ((d)–(g)) and cND-Fe3O4 ((h)–(k)). *A* = prophase; *B* = metaphase; *C* = anaphase; *D* = telophase; *E* = chromosomal break; *F*=cytoplasmic bridge; *G* = disturbed anaphase; *H*= laggard; *I* = sticky anaphase; *J* = scattered anaphase; *K* = prophase nuclei with micronucleus in interphase; *L* = binucleate cells.

Fe_3_O_4_ and cND exhibit their differentiating consequences for mitosis and other cyto-genotoxic lists examined here. In observation with each one of the convergences of Fe_3_O_4_ contemplated, immaterial mitodepressive and cyto-genotoxic nature was shown by each one of the groupings of cND. (Tables 4 and 5; Figure 4) represent the images of different chromosomal aberrations observed in *Allium cepa* L. Figure 4(a) is prophase; Figure 4(b) is metaphase; Figure 4(c) is anaphase; Figure 4(d) is telophase; Figure 4(e) is chromosomal break; Figure 4(f) is cytoplasmic bridge; Figure 4(g) is disturbed anaphase; Figure 4(h) is laggard; Figure 4(i) is sticky anaphase; Figure 4(j) is scattered anaphase; Figure 4(k) is prophase nuclei with micronucleus in interphase; Figure 4(l) is binucleate cells.

The untreated root tip cells demonstrated a mitotic file of 71.3 ± 2.2% (Table 1). However, a dose-dependent effect on the mitotic index was noted for Fe_3_O_4_ and Fe_3_O_4_-cND. In particular, the mitotic records were observed to be 58.07 ± 1.7, 37.8 ± 1.2, and 28.6 ± 0.8% upon presentation to 5, 10, and 20 *μ*g·mL^−1^, respectively. Remarkably, diminishes in MI were irrelevant at these convergences of cND with estimations of 68.3 ± 2.0, 65.7 ± 1.9, and 59.0 ± 1.7, separately, as it may be seen in Table 4 very well. The ameliorative impact of cogathered effects is shown by low (5 *μ*g·mL^−1^) and moderate (10 *μ*g·mL^−1^) concentrations of cND-Fe_3_O_4_. This shows if unintentionally discharged into condition, cND-Fe_3_O_4_ would be alright for biotic life to the most extreme grouping of 10 *μ*g·mL^−1^.

The observed cogathered cyto-genotoxic outcomes agree with comparable prior investigations, where cooxide nanoparticles were assumed to destroy the entire cell digestion and phases of cell division for the most part by blocking water channels through adsorption as well as by affecting hereditary material by causing different sorts of chromosomal mutilation.

Anjum et al. [29] previously explained the expanded amount of aggregate chromosomal abnormalities with increasing test groupings of different nanoparticles. Sticky chromosomes are primarily thought of as a type of chromatid aberration that results from the corruption or depolymerization of chromosomal DNA [27]. Instead, sticky chromosomes were among them, and they were observed as frequently as possible throughout the anaphase and telophase stages of mitosis in the root tips of the A. cepa plant (onion). In summary, the minor alterations in MI with a moderate concentration (10 g·mL^−1^) of cND-Fe3O4 also support the notion that this compound had a significant interference with the normal progression of mitosis, primarily due to its inability to prevent cells from entering the prophase and obstruction of the mitotic cycle during the entomb stage restraining DNA/protein combination.

Moreover, compared to 20 *μ*g·mL^−1^ of cND-Fe_3_O_4_ and 5, 10, and 20 *μ*g·mL^−1^ of Fe_3_O_4_, 10 *μ*g·mL^−1^ of cND-Fe_3_O_4_ presents insignificant and infrequent chromosome deviations (for example, stickiness, breaks, irritated, and dissipated metaphase); therefore, these results strongly support the ecological benevolent nature of the cND-Fe_3_O_4_ nanocomposite (Table 5) .

## 4. Conclusions

The novel ND/Fe_3_O_4_ nanocomposite has been effectively arranged by the in-situ technique. The produced ND/Fe_3_O_4_ nanocomposite shows a magnetic hysteresis loop, indicating it might be used as a possibility to obtain a bright magnetically separable photo-catalyst for the expulsion of natural contamination from water. The weight level of Fe_3_O_4_ is 28% and that of ND is 72% in the ND/Fe_3_O_4_ composite. The ND/Fe_3_O_4_ composite also shows electrical conductivity of the order of 26 times compared to ND soot. We are also in the process of preparing the ND/Fe_3_O_4_ composite cross-breed nanofluids for warm applications. Nonetheless, chromosome variations (for example, stickings, prospect, and dispersed metaphase) are inconsistent for the cND-Fe_3_O_4_ nanocomposite at a convergence of 10 g·mL^−1^, in contrast to 20 g·mL^−1^ of cND-Fe_3_O_4_ and 5, 10, and 20 g·mL^−1^ of Fe_3_O_4_, clearly indicating that at this concentration, cND-Fe_3_O_4_ is environmentally ecofriendly.

## Figures and Tables

**Table 1:** X-ray diffraction positions (2*θ*) and the interplanar spacing values (*d* hkl) of the Fe_3_O_4_ sample (blue color line).

2*θ*	*d* (Å)	(hkl)	Phase (microstructure)
30.09	2.96	(220)	Magnetite (Fe_3_O_4_)
35.49	2.52	(311)	Magnetite (Fe_3_O_4_)
43.28	2.09	(400)	Magnetite (Fe_3_O_4_)
56.99	1.61	(511)	Magnetite (Fe_3_O_4_)
62.62	1.48	(440), (214)	Magnetite + *α*−Fe_2_O_3_
74.02	1.28	(533)	Magnetite (Fe_3_O_4_)

**Table 2:** X-ray diffraction positions (2*θ*) and the interplanar spacing values (*d* hkl) of the ND sample (black color line).

2*θ*	*d* (Å)	(hkl)	Phase (microstructure)
43.58	2.068	(111)	Cubic nanodiamond
74.63	1.267	(220)	Cubic nanodiamond

**Table 3:** X-ray diffraction positions (2*θ*) and the interplanar spacing values (*d* hkl) of the ND-Fe_3_O_4_ sample (red color line).

2*θ*	*d* (Å)	(hkl)	Phase (microstructure)
30.09	2.96	(220)	Magnetite (Fe_3_O_4_)
35.49	2.51	(311)	Magnetite (Fe_3_O_4_)
43.58	2.068	(111)	Cubic nanodiamond
43.28	2.09	(400)	Magnetite (Fe_3_O_4_)
56.99	1.61	(511)	Magnetite (Fe_3_O_4_)
62.62	1.48	(440), (214)	Magnetite + *α*-Fe_2_O_3_
74.02	1.28	(533)	Magnetite (Fe_3_O_4_)
74.63	1.267	(220)	Cubic nanodiamond

**Table 4:** mitotic index and percent phase indices of prophase, metaphase, anaphase, and telophase stages in *Allium cepa* root exposed to Fe_3_O_4_, cND, and cND-Fe_3_O_4_.

Concentrations (*μ*g mL^−1^L)	Nanoparticles	Mitotic index (mean ± SE)	Prophase (%)	Metaphase (%)	Anaphase (%)	Telophase (%)
Control	—	71.3 ± 2.2	62.3	4.03	2.4	3.7

5	Fe_3_O_4_	58.07 ± 1.7^a^	54.2	3.5	2.0	3.1
	cND	68.3 ± 2.0^b^	59.0	3.9	2.1	3.5
	cND-Fe_3_O_4_	67.5 ± 2.0^bc^	58.7	3.6	2.4	3.7

10	Fe_3_O_4_	37.8 ± 1.2^a^	43.8	3.0	1.7	2.7
	cND	65.7 ± 1.9^ab^	55.3	3.3	2.04	2.9
	cND-Fe_3_O_4_	63.8 ± 1.9^ab^	56.1	2.8	2.3	3.04

20	Fe_3_O_4_	28.6 ± 0.8^a^	32.6	1.7	1.77	2.5
	cND	59.0 ± 1.7^ab^	51.8	2.8	2.1	2.8
	cND-Fe_3_O_4_	51.7 ± 1.5^ab^	44.07	3.05	1.2	3.2

(*n* = 3). Significant variations within the same concentrations are aVs. control; bVs.Co; cVs.cND. Footnote: the mitotic index and percent phase indices of prophase, metaphase, anaphase, and telophase stages in *Allium cepa* root meristematic cells were exposed to various concentrations of cobalt oxide (Fe_3_O_4_), carboxylated nanodiamond (cND), and cND-Fe_3_O_4_. 1000 cells were scored per treatment group.

**Table 5:** Quantitative measurements of occurrences of various chromosomal aberrations noticed in *Allium cepa* root exposed to Fe_3_O_4_, cND, and cND-Fe_3_O_4_.

Concentrations (*μ*g mL^−1^L)	Nanoparticles	Chromosomal breaks (%)	Chromosomal bridges (%)	Sticky chromosomes (%)	Laggard chromosomes (%)	Disturbed anaphase/metaphase (%)
Control	—	0 ± 0	0 ± 0	0 ± 0	0 ± 0	0 ± 0

5	Fe_3_O_4_	35.5 ± 2.5	41.2 ± 2.8	26.7 ± 1.8	8.8 ± 0.6	7.4 ± 0.5
	cND	3.2 ± 0.2^a^	4.6 ± 0.3^a^	0 ± 0	0 ± 0	1.3 ± 0.1^a^
	cND-Fe_3_O_4_	8.5 ± 0.6^abc^	14.3 ± 1.0^abc^	4.4 ± 0.3^abc^	0 ± 0	3.7 ± 0.3^abc^

10	Fe_3_O_4_	44.7 ± 3.1	57.7 ± 4.0	33.7 ± 2.3	14.5 ± 1.0	13.5 ± 0.9
	cND	5.5 ± 0.4^a^	6.6 ± 0.4^a^	1.4 ± 0.1^a^	1.3 ± 0.1^a^	3.5 ± 0.3^a^
	cND-Fe_3_O_4_	17.3 ± 1.2^abc^	21.4 ± 1.5^abc^	5.7 ± 0.4^abc^	3.5 ± 0.2^abc^	6.0 ± 0.4^abc^

20	Fe_3_O_4_	58.8 ± 4.1	69.4 ± 4.8	47.9 ± 3.3	21.4 ± 1.5	20.5 ± 1.4
	cND	7.1 ± 0.5^ab^	8.5 ± 0.6^a^	2.6 ± 0.2^a^	4.0 ± 0.3^a^	5.3 ± 0.4^a^
	cND-Fe_3_O_4_	26.4 ± 1.8^abc^	27.8 ± 2.0^abc^	11.5 ± 0.8^abc^	16.6 ± 1.2^abc^	9.7 ± 0.7^abc^

(*n* = 3). The significant variance is shown within the same concentration: aVs. control; bVs.Co; cVs.cND.

## Data Availability

All relevant data are within the paper and their supporting information files.
